# Sunstroke—Asthma

**Published:** 1890-02

**Authors:** Wm. Steinrauf

**Affiliations:** St. Charles, Missouri


### SUNSTROKE.—ASTHMA.

Wm. Steinrauf, M. D., St. Charles, Missouri.

Mrs. K., aged fifty-four years, was afflicted with *coup de soleil* more than eighteen years ago, and her regular allopathic adviser sent for without delay. He immediately had her stripped of all her clothing and plunged into ice-cold water for several hours. She regained consciousness after a lapse of ten hours and in a few days was able to attend to her household duties again. With the exception of a clogged-up sense of the nostrils and a slight constriction of the chest she appeared to be all right. Not more than four weeks after this attack she began to have regular asthmatic spells, occurring about every two or three weeks, for which her doctor gave her hypodermic injections of Morphia. These spells have now been troubling her with increased virulence since the day she was taken with sunstroke and the injections kept up the same length of time.

When we saw her about six months ago, she was just having one of her characteristic "spells" and a pitiful sight it was. We found her propped up in bed, breathing with great rapidity, unable to speak, with a pulse of one hundred and ten, and the cold perspiration running down her face in great streams. As this was the worst attack she had ever had she thought she would die, in which belief I was inclined to coincide. Herself and husband attributed all her troubles to the heroic treatment she had received eighteen years ago for sunstroke. In all these long and weary years her physician had failed to do her any good; simply injecting Morphia as the attacks came on. Could Homœopathy do her any good? Our divine art is very much decried in the State of Missouri, and it was with misgivings that she sent for me. After hearing her story and getting her symptoms, I left Sac. lac., and went home to study the case, promising to send the remedy in two hours. The most prominent symptom was a continual desire to move. On the strength of this and several other Rhus-tox. symptoms, I gave the messenger three doses of this remedy to be taken, a powder every hour, and afterward to resume the Sac. lac. A terrible diarrhœa came on the next day which I thought best not to disturb. It left after a few days, and Mrs. K. has since been free of her spells, and considers herself well. I have reason to believe that she is correct in her belief. Rhus-tox. (Swan) CM. did the work.

